# Drug Consumption in Conflict Zones in Somalia

**DOI:** 10.1371/journal.pmed.0040354

**Published:** 2007-12-11

**Authors:** Kamaldeep Bhui, Nasir Warfa

## Abstract

The authors discuss the public health implications of a new study on the use of khat and other drugs by Somali combatants.

Conflict within and between countries based on tribes, clans, religion, and political ideology is well described worldwide. War is known to contribute to a higher burden of mental health problems among specific individuals who experience trauma as well as among those living in or near to conflict zones. The mental health burden is mostly recognised as post-traumatic stress disorder [1–3]. However, during conflict there is also significant disability from common mental health problems such as depression, anxiety, and substance misuse, which are linked with the poverty associated with economic fragmentation and with a reduction of basic security functions and safety.

## Substance Use and Conflict in East Africa

In a new cross-sectional study published in this issue of *PLoS Medicine*, Michael Odenwald and colleagues interviewed 8,723 Somali combatants to assess their use of khat and other drugs [4]. In total, 36.4% (99% confidence interval, 19.3%–57.7%) of respondents reported khat use in the week before the interview.

The excessive use of substances for self-medication in conflict zones may be a form of coping with traumatic memories of atrocities, but the choice of drug and the patterns of use have not been well studied amongst former or current combatants in conflict zones in East Africa. The routes by which drugs, for example khat and alcohol, find their way into East Africa and specifically Somalia are well known, yet preventive actions appear not to actively address these supply routes. In part, the resistance to cutting off the supply routes comes from those who rely on the income from selling these drugs. In addition, khat and other drugs are products of their culture, and therefore, to challenge their use or prohibit them would be to attack a cultural practice and undermine traditions. By analogy, alcohol is the cause of enormous morbidity and mortality in the West, yet it is not outlawed in part because its use is culturally sanctioned by Western culture.

Research on mental health problems and drug use in war zones is essential to fully inform health initiatives. However, in zones where security functions are absent, and in active war zones with multiple security forces, those promoting public health and research will find their work challenging and dangerous.

**Linked Research Article**

This Perspective discusses the following new study published in *PLoS Medicine:*

Odenwald M, Hinkel H, Schauer E, Neuner F, Schauer M, et al. (2007) The consumption of khat and other drugs in Somali combatants: A cross-sectional study. PLoS Med 4(12): e341. doi:10.1371/journal.pmed.0040341

Having interviewed military personnel in Somali, Michael Odenwald and colleagues conclude that drug-related problems, mainly relating to the use of khat, have reached proportions formerly unknown to the country.

## Strengths and Weaknesses of the New Study

An innovation in Odenwald and colleagues' study was to engage local non-governmental organisations working in conflict zones in both North and South Somalia; these provided the interviewers for the study. The researchers used a convenience sample of former and current combatants recruited through their militia units, and interviewed those who had been commanded by senior officers to participate in the conflict. The authors report that many interviewees were keen to participate in the study, as they hoped that it would lead them to find help and a pathway to recovery. This type of study raises a number of ethical concerns. For example, which local governing bodies are responsible for ensuring ethical conduct of research in war zones? Did the militia men participate in the study willingly if they were ordered by superior officers?

Another innovation in Odenwald and colleagues' study is that it distinguished between cultural practice and abnormal use of khat, and it also investigated the use of other substances. The researchers found that khat is both perceived to be used and is actually used by a significant majority of young former and current combatants, and is by far the most used drug in East Africa compared with hashish, tablets of any sort, alcohol, inhalants, and bangi (hemp) seeds. They also found that while khat is equally used in the North and South of the country, excessive use of most substances was found in the South of Somalia, a conflict zone. This suggests that conflict has been associated with increased substance misuse.

Odenwald and colleagues compare their finding of equivalent rates of khat use in the North and South with an earlier epidemiological study that showed a lower use in the South [5]. They conclude that this discrepancy indicates that khat use has significantly increased in the South since the destruction of the Somali state in 1991. However, it is important to bear in mind that they used a convenience sample, which may have attracted participants with substance use problems who wanted help, and therefore this method may yield higher estimates of prevalent khat use.

## Khat and Mental Illness

Khat is reported to be an amphetamine-like substance; when used excessively it increases the risk of mental illness. When used in moderation, it appears to have more of a social function akin to alcohol use. In Europe, a moral panic emerged about khat use and misuse and how it may contribute to disability [6]. Even in the European Union there are contradictions; the use of khat is illegal in most EU countries but not in the United Kingdom or in Holland. Khat is also illegal in Canada and America but is smuggled into these countries frequently. In our previous cross-sectional studies in the UK, we found associations between khat use and mental illness [3,7]. Yet the challenge to the legal use of khat has been made mostly on evidence from case reports and anecdotes [6]. Prospective studies are needed.

What Odenwald and colleagues' new study makes clear is that health prevention work in conflict zones should emphasise both mental and physical health problems; certainly these problems can be tackled through addressing sources of conflict and reconciling them through political means. Little has been said about managing drug trafficking more actively in order to prevent young and vulnerable people being exposed to psychoactive drugs that could contribute to disability and violence. Similar issues emerge when considering the use of cannabis or heroin from plantations in developing countries that rely on the drug trade to fuel the economy. In Europe, price rises are seemingly the only way to curb excessive levels of use of alcohol and nicotine.

The extent to which the dramatic rise in khat usage in Somalia perpetuates the Somali conflict or how the trade of khat finances international terrorism is debatable. However, what is clear is that khat misuse significantly exacerbates poverty levels and has a negative impact on the living standard of the Somali people, who already live in one of the poorest countries of the world. According to the 2004 UK Department for International Development country engagement plan for Somalia, a significant number of people in Somalia live in extreme poverty [8]. For example, 60% of the population were living on below US$1 a day, and per capita GDP is reported to have gone down from US$280 in 1989 to US$266 in 2002 [8]. And yet up to 80% of the Somali male population spends around US$4 a day on khat each day. How could one expect to reduce poverty levels and improve the quality of life in poor nations such as Somalia when the international community is reluctant to address the very problem that perpetuates various social, economic, and political instabilities? As we understand the situation, khat, which is used by around 10 million people in East Africa, is currently not of concern to the UN and its various agencies, while alcohol and other polydrug use in the West remain a higher priority.

The challenge facing Somalia and other conflict zones is that it is young people who are most vulnerable to developmental insults, which can lead to long-lasting and, in some instances, permanent mental health and physical health problems. Yet it is these very people who are likely to be recruited for warfare and are active in conflict zones; specifically young men exposed to drug use and violence, who will then have the most difficulty adjusting to a life free of violence. These issues warrant further investigation with the engagement of clan leaders and the non-governmental sector if peace and reconciliation are to be realised and if economic prosperity and the UN Millennium Goals [9] are to be achieved by all sectors of a wartorn society.
